# Purification of Lectin from Larvae of the fly, *Musca domestica*, and in Vitro Anti-Tumor Activity in MCF-7 Cells

**DOI:** 10.1673/031.010.14124

**Published:** 2010-09-29

**Authors:** X. Cao, Z. Huo, M. Lu, D. Mao, Q. Zhao, C. Xu, C. Wang, B. Zeng

**Affiliations:** Ministry of Education, Key Laboratory of Food Nutrition and Safety, School of Food Science and Biotechnology, Tianjin University of Science and Technology, Tianjin 300457, P. R. China

**Keywords:** *Musca domestica* larvae, Lectin, Apoptosis, Anti-tumor activity

## Abstract

A new lectin was purified from larvae of the fly, *Musca domestica* L. (Diptera: Muscidae) (MLL-2, 38 kDa) using affinity chromatography and HPLC. Anti-tumor activity of MLL-2 was demonstrated by its inhibition of proliferation of human breast cancer (MCF-7) cells in a time-and dose-dependent manner. The results of acridine orange staining indicated that MLL-2 caused apoptosis in MCF-7 cells. DNA fragmentation in MCF-7 cells has been detected by TUNEL. Flow cytometric analysis also demonstrated that MLL-2 caused dose-dependent apoptosis of MCF-7 cells through cell arrest at G2/M phase. The MLL-2 induced a sustained increase in concentration of intracellular free calcium. Western blot revealed that MLL-2 induced apoptosis in MCF-7 cells was associated with typical apoptosis proteins in the mitochondrial pathway. In addition, the caspase-3 activity in MCF-7 cells treated with MLL-2 for 48 hours was significantly increased compared to controls (407.4 ± 3.0 vs. 1749.2 ± 6.0, P <0.01). Since MLL-2 induced apoptosis in MCF-7cells the mitochondrial pathway may be the main pathway of antitumor activity.

## Introduction

Lectins are proteins or glycoproteins with the ability to bind, selectively, free or conjugated saccharides in a reversible way by two or more binding sites (Sharon 1993) and are ubiquitous in all forms of living matters, including bacteria, plants, and animals (Rhodes 1999). They recognize sequences of two or more saccharides with specificity towards both inter-residue glycosidic linkages and anomeric configuration, so that they have anti-bacterial and anti-tumor effects by recognizing residues of glycoconjugates on the surface of cells (Glatz et al. 2004; Simone et al. 2006).

Apoptosis, a type of programmed cell death, is an active process. It is a normal component of the development and health of multicellular organisms. The study of apoptosis is an important field of biological inquiry since a deficiency or an excess of apoptosis is one of the causes of cancers, autoimmune disorders, diabetes, Alzheimer's, organ and bone marrow transplant rejection, and many other diseases. There are some pathways that lead to apoptosis, p53, Bcl-2 and BAD proteins play central roles in the mitochondrial pathway. P53 is a tumor suppressor protein that in humans is encoded by the TP53 gene (Matlashewski et al. 1984; Isobe et al. 1986; Kern et al. 1991). In a normal cell p53 is inactivated by its negative regulator. Upon DNA damage or other stresses, various pathways will lead to the dissociation of sp53. Once activated, p53 will induce a cell cycle arrest to allow either repair and survival of the cell or apoptosis to discard the damaged cell. As such, p53 has been described as “the guardian of the genome”. Bcl-2 family proteins regulate and contribute to programmed cell death or apoptosis. It is a large protein family and all members contain at least one of four bcl-2 homology domains. Certain members are pro-apoptotic (BAD, Bax, Bak and Bok among others) or antiapoptotic (including Bcl-2 proper, Bcl-xL, and Bcl-w, among others).

Recently, extensive studies have revealed that a number of lectins from plants can be used for prevention and/or treatment of cancer. For example, mistletoe lectins have been shown to have therapeutically active anti-cancer effects on cancer (Franz 1985). Lectins from mushroom species including *Agaricus bisporus, Boletus satanus, Flamulina velutipes, Ganoderma lucidm* have antitumor activities (Wang et al. 1998). In addition, some studies have shown that animal lectins, for example, *Drosophila* C-type lectins (Jingqun et al. 2007), *Sarcophaga* lectin (Itoh et al. 1986), and a sea urchin lectin (Masao 2006), play important roles in controlling immune responses and antitumor activities in vitro and in vivo. But relatively few studies have been conducted on lectins from *Musca domestica* L. (Diptera: Muscidae).

Although some studies have tested lectins for potential anti-tumor effects, the mode of action of lectins has not been elucidated in detail, and there is no report on the effect of MLL-2 purified from *M. domestica* larvae on human breast cancer MCF-7 cells. Thus, the aim of the present study was to evaluate the growth inhibition effects of MLL-2 purified from *M. domestica* larvae against MCF-7 cells and its apoptosis-inducing activity, with special emphasis on its mode of action.

## Materials and Methods

### Isolation and Purification

*Musca domestica* was obtained from the Tianjin Sanitation and Epidemic Prevention Station, Tianjin, P.R.China. Approximately 100 g larvae were ground with a mortar at 4° C by adding 50 ml buffered insect saline (10 mM Tris/HCl, 130 mM NaCl, 5 mM KCl, pH 7.4) and 1g phenylthiocarbamide (Sigma, www.sigmaaldrich.com). The whole body extract was extracted for 30 minutes followed by centrifugation (8000 rpm, 20 min at 4° C). The resulting supernatant was the crude body extract.

The crude extract was mixed with 20 ml Sepharose-4B with slow stirring for at least 1.5 h at 4° C. The mixture was put into a 1.5×22 cm column (Bio-Rad, www.bio-rad.com) and washed with 200 ml saline with a flow rate of 1 ml min^-1^ until no protein was detected in the elute by monitoring the absorbance at 280 nm. Then attached proteins were subsequently eluted by 0.2 M D-galactose

The fractions were pooled in an ultrafiltration cell (8050, Millipore, www.waters.com) and concentrated by ultrafiltration with a 3 kDa membrane (Polyethersulfone, Biomax PB, Millipore). Samples were freeze-dried before storage (Cao et al. 2010).

### Purification by HPLC

Lectin samples were applied to an HPLC column (TSK gel Super SW3000, 4.6 mm × 30 cm, Tosoh, www.tosoh.com) at a lectin concentration of 1 mg ml^-1^. Phosphate buffered solution at pH 6.4 was used as elution buffer at a flow rate of 1 ml min^-1^ successively and the absorbance at 280 nm was monitored.

### Haemagglutinating assay

To measure haemagglutinating activity of lectin, 25 µl of a serially diluted sample was mixed with 25 µl 2.0% trypsinized red blood cells of rabbits. The suspension containing 1% (w/v) bovine serum albumin was incubated for 1 h at 37° C in a well of a V-bottomed micro-titer plate. The haemagglutinating titer, defined as the reciprocal of the highest dilution exhibiting haemagglutination, was one haemagglutinating unit. The specific activity was calculated as the number of haemagglutinating units per mg protein (Wang et al. 2000).

### Cell lines and culture

Human breast cancer (MCF-7) cells and human lung fibroblasts (HLF) cells were obtained from Tianjin Medical University. MCF-7 cells were cultured in RPMI-1640 medium (GIBCO, www.lifetech.com) supplemented with 10 % heat inactivated (56° C, 30 min) fetal calf serum (Beijing Yuanheng Shengma Research Institution of Biotechnology, Beijing, China), 100 U ml^-1^ penicillin (GIBCO) and 100 µg ml^-1^ streptomycin (GIBCO). HLF cells were routinely grown in Dulbecco's Modified Eagle's Medium (GIBCO) containing 10% fetal bovine serum, 100 U ml^-1^ penicillin and 100 µg ml^-1^ streptomycin. All cells were cultivated at 37° C with 5% CO_2_.

### Growth inhibition assay

Cytotoxic effects on the growth and viability of cells were determined using tetrazolium dye (MTT) assay as previously described (Horiuchi et al. 1988). Briefly, MCF-7 cells and HLF cells at concentration of 1.5×10^5^ cells ml^-1^ were seeded in the 96-well plates (COSTAR, www.corning.com/lifesciences). After 24 h, the cells were treated with various concentrations (6.5–250 µg ml^-1^) of MLL-1 or MLL-2 for 24, 36 and 48 h respectively. MTT reagent (20 µl) was added to each of the 100 µl culture wells. The MTT reagent was prepared at 5 mg ml^-1^ in PBS, filter sterilized and stored in the dark at 4° C for a maximum of 1 month. After incubation for 4 h at 37° C, the water-insoluble formazan dye formed was solubilized by adding 150 µl DMSO to the culture wells. The plates were shaken mildly for 10 min at room temperature and optical density of the wells was determined using ELISA microplate reader at a test wavelength of 570 nm and a reference wavelength of 690 nm. Control cells were grown under the same conditions without addition of MLL-1 or MLL-2. Inhibition(%) was calculated according to the method: [(C-T)/C]×100, in which C is the average OD of control cells group and T stands for the average OD of MLL-1, MLL-2 treated group.

### Observation of morphological changes

Acridine orange (AO) staining is a routine diagnostic technique for apoptotic cells morphology. MCF-7 cells were seeded on slides, and placed in culture-plates. After 24 h, the cells were treated with MLL-2 (100 µg ml^-1^) for 0, 24, 48 and 72 h, respectively. The cells were fixed with 70% ethanol and slides were washed three times with PBS. AO (8.5 µg ml^-1^) was added to each slide. The slide was incubated at room temperature under reduced light for 20 min. Fluorescence was detected by a fluorescence microplate reader using filter sets, excitation at 485 nm, emission at 530–640 nm (Petit et al. 1994; Gallet et al. 1995).

### Apoptosis TUNEL Assay

Terminal deoxynucleotidyl transferase-mediated dUTP-biotin nick-end labeling (TUNEL) is an *in situ* method for detecting the 3′-OH ends of DNA exposed during the internucleosomal cleavage that occurs during apoptosis. Incorporation of biotinylated dUTP allows detection by immunohistochemical procedures. This technique consists of pretreatment of cells with protease and then incorporation of a labeled oligo (dU) into the DNA breaks with terminal deoxy-transferase. Finally, dU is visualized with peroxidase. The morphologic features were visualized by light microscopy. The TUNEL reaction is highly specific and only apoptotic nuclei are stained (Gavrieli et al. 1992).

### Flow cytometric analysis of the cell cycle and ratio of apoptotic cells

The effects of MLL-2 on proliferation of cells was evaluated by measuring the distribution of the cells in the different phases of the cell cycle by flow cytometry. Cells were treated with 100 µg ml^-1^ MLL-2 for the indicated times and harvested by centrifugation at 1,000 rpm for 5 min at room temperature. Resuspended cells were pellet in 1 ml of cold PBS, and cells were fixed by adding cold 75% ethanol at 4° C for 18 h. Fixed cells were washed with PBS. 100 µl of 200 µg ml^-1^ DNase-free, RNase was added and cells were incubated at 37° C for 30 min, and resuspended in a staining solution containing propidium iodide (1 mg ml^-1^) and incubated at room temperature for 5–10 min in the dark. The cell suspensions were placed in 12 × 75 falcon tubes and analyzed on a fluorescenceactivated cell sorter flow cytometer (Coulter Epics XL, Beckman Coulter, www.beckmancoulter.com). Results shown are from three different experiments.

### Measurement of intracellular free calcium [Ca^2+^]_i_

Briefly, MCF-7 cells were plated on slides at a density of 2×10^6^ cells ml^-1^, and incubated with 5% CO_2_ at 37° C for 24 h. After cells were stimulated by 100 µg ml^-1^ MLL-2 for 4, 8, 12 h, and loaded with Fluo-3/AM (5 µmol L^-1^, Sigma) at 37° C under reduced light for 60 min. Intracellular free calcium [Ca^2+^]i was measured by laser scanning confocal microscopy (LSCM, Leica, www.leicamicrosystems.com).

### Western blot analysis of p53, Bcl-2 and Bad expression

Western blot analysis of p53, Bcl-2 and Bad protein from the lysates of MCF-7 cells treated with 100 µg ml^-1^ MLL-2 for 4, 8, 12, 24 and 48 hours were mixed with an equal volume of 1 × SDS-PAGE sample buffer, heated at 100° C for 5 min and loaded onto a 10% SDS Polyacrylamide gel. The Nc membrane was blocked with 5% non-fat milk in phosphate-buffered saline containing 0.05% Tween-20 prior to antibody treatments. A primary antibody (Santa Cruz Biotechnology, www.scbt.com) was then added to the solution which binds to its specific protein. A secondary antibodyenzyme conjugate, which recognizes the primary antibody, is added to find locations where the primary antibody bound. The protein of interest was visualized by enhanced chemiluminescence reagents (ECL kit, Amersham Pharmacia Biotech, www.apbiotech.com). The protein side of the membrane was exposed to X-ray film.

**Figure 1.**
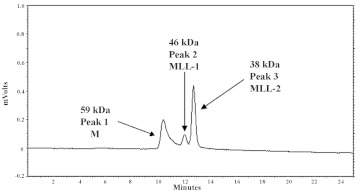
MLL (1 mg ml^-1^) were applied on a TSK gel column (Super SW3000 4.6 mm×30cm). Phosphate buffered solution pH 6.4 was used as elution buffer with a flow rate of 0.1 ml min. The HPLC spectrograms of extracts from MLL: peak1 is 59 kDa (M), peak2 is 46 kDa (MLL-1), peak3 is 38 kDa (MLL-2). High quality figures are available online.

### Measurement of caspase-3 activity

Cells treated with 100 µg ml^-1^ MLL-2 for 12, 24, 36, 48 hours, respectively. Cultured MCF-7 cells were lysed with a lysis buffer (50 mM Hepes, 100 mM NaCl, 0.1% CHAPS, 1 mM EDTA, 10 mM DTT, 10 % Glycerol, Sigma). The soluble fraction of the cell lysate was assayed for caspase-3 activity using Ac-DEVD- *p*NA substrate (Sigma). After incubation for two hours at 37° C with 5% CO_2_ the intensity of the color reaction was measured using a microplate reader at 405 nm.

### Statistical analysis

The experiments were repeated three times and the mean values from both treated and untreated groups were analyzed by a twotailed unpaired *t*-test. The level of *P*<0.05 was considered to be statistically significant.

## Results

### Purification results

After affinity chromatography using Sepharose-4B and further separation on a TSK gel Super SW3000 HPLC column, three peaks were observed with molecular weights of 59 kDa (M), 46 kDa (MLL-1) and 38 kDa (MLL-2) (Figure 1). The 59 kDa (M) peak did not exhibit haemagglutinating activity. However, the MLL-1 and MLL-2 peaks exhibited haemagglutinating activity and the MLL-2 haemagglutinating activity was higher than MLL-1 haemagglutinating activity.

### Inhibitory effect of MLL-1, MLL-2 on MCF-7 cells growth

**Figure 2.**
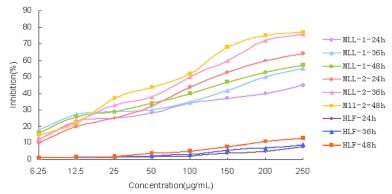
Cytotoxic effect of MLL-1 and MLL-2 in MCF-7 cells. Cells were cultivated in RPMI 1640 medium with indicated concentrations of MLL-2 for 24, 48 and 72 h, respectively. HLF cells were used to examine cytotoxic effect of MLL-1, MLL-2 on normal cells of human beings. The viability of cells was determined by the MTT assay. The cytotoxicity of each reagent was expressed as an IC50 value. Each value represents mean±S.D. of three independent experiments. High quality figures are available online.

MLL-2 inhibited the viability of MCF-7 cells in a time-and dose-dependent manner (Figure 2). HLF cells were used to examine cytotoxic effect of MLL-1, MLL-2 on normal human lung fibroblast cells, and viability of these cells was not significantly affected(<15% inhibition). 48 hours of continuous exposure to different doses of MLL-1 or MLL-2 resulted in cessation of cell growth followed by significant cell death. The IC50 of MLL-1 was 200 µg ml^-1^ and the IC50 of MLL-2 was 100 µg ml^-1^ for MCF-7 cells at 48 hours. MLL-2 had higher inhibitory effects on MCF-7 cells growth, thus MLL-2 were used for the following experiments.

**Figure 3.**
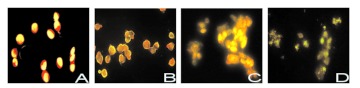
Effect of AO staining by MLL-2 on MCF-7 cells. (A) Control group. (B) After 24 h, the morphology of cells still were still integrated, and budding. (C) For 48 h group, the nuclear gradually break. (D). After 72 h, the nuclear completely break, and green/yellow fluorescent AO staining indicates fully condensed, the nucleus into small apoptotic bodies. Original magnification×400. High quality figures are available online.

### Morphological changes

AO staining was used to study the apoptosis morphology of MCF-7 cells that were treated with 100 µg ml^-1^ MLL-2. In controls, the cytoplasm of MCF-7 cells is fluorescent red, and the nucleus is fluorescent yellow (Figure 3A). After 24 hours, the the cells were still intact and budding (Figure 3B). After 48 hours, the nuclei gradually disintegrated (Figure 3C). After 72 h, the nuclei were completely disintegrated, and green/yellow fluorescent AO staining indicated that the nuclei had fully condensed into small apoptotic bodies (Figure 3D).

### Result of TUNEL staining in MCF-7 cells

**Figure 4.**
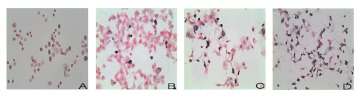
The TUNEL method with peroxidase labeling. Effect of MLL-2 on MCF-7 cells. (A) Control group. (B) 24 h group presented the similar apoptosis compared with control group, almost all cell was pink. (C) 48 h group, MLL-2 significantly increased the apoptosis of MCF-7, as evidenced by the more TUNEL-positive cells (blue purple). (D) After 72 h, majority of cell were blue purple (apoptotic cells). Original magnification×200. High quality figures are available online.

To determine the apoptosis of MCF-7 cells in different groups, TUNEL staining was used. The results of TUNEL staining are shown in Figure 4. The 24 hour group showed similar apoptosis compared with control group, almost all cells were pink (Figure 4A and Figure 4B). In the 48 hour group, MLL-2 increased apoptosis of MCF-7 cells, as evidenced by the more TUNEL-positive cells (blue purple) (Figure 4C). After 72 h, majority of cell were blue-purple (apoptotic cells).

### Effect of MLL-2 on MCF-7 cells cycle and ratio of apoptotic cells

Flow cytometry was used to examine whether MLL-2 could interfere with the cell cycle of MCF-7 cells stained with propidium iodide. The results of the cell cycle distribution are presented in Table 1. After MCF-7 cells were treated with 100 µg ml^-1^ of MLL-2 for various times, an arrest of the cell cycle at the G_2_/M phase (from 15.00±0.20% to 30.40±0.25%, P<0.01) and significantly increased apoptotic rates (from 5.38±0.04% to 17.70±2.24%, P<0.01) were observed.

**Table 1.**
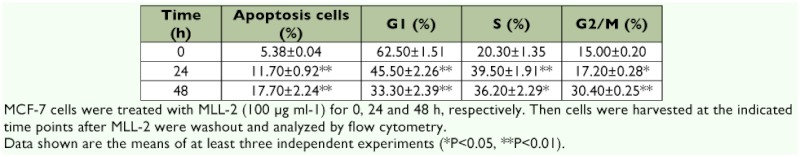
Time course analysis of the cell cycle distribution in MLL-2 treated MCF-7 cells.

**Figure 5.**
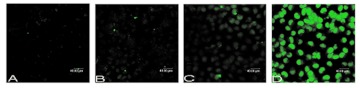
Cells were treated with MLL-2 for 4, 8 and 12 h, respectively. The fluorescence intensity of Fluo-3/AM in the MCF-7 cells were 67.67 (B), 128 (C) and 223.38 (D) while the control group was 28.33 (A). The result demonstrated that the intracellular calcium concentration in MCF-7 cells increased with a statistically significant difference. High quality figures are available online.

### Effect of MLL-2 on the expression of intracellular free calcium ([Ca^2+^]_i_)

**Figure 6.**
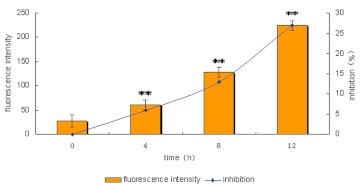
The fluorescent intensity of [Ca^2+^]_i_ is a time-dependent manner. With the increase of [Ca^2+^]_i_, inhibition of cells significantly increase (**P<0.01). High quality figures are available online.

The production of [Ca^2+^]_i_ increased rapidly in the MCF-7 cells after stimulation with 100 µg ml^-1^ MLL-2 for 4, 8 and 12 hours. The fluorescent intensity of Fluo-3/AM in the MCF-7 cells was 67.67 (Figure 5B), 128 (Figure 5C) and 223.38 (Figure 5D) while the control group was 28.33 (Figure 5A). Laser scanning confocal microscopy showed that compared to the control group, the average intensity of [Ca^2+^]_i_ fluorescent signal in MCF-7 cells treated with MLL-2 increased significantly (P <0.01). Fluorescent signal intensity was time-dependent (Figure 6).

**Figure 7.**
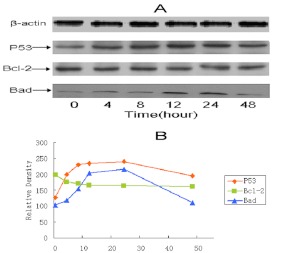
Effect of MLL-2 on the expression of p53, Bcl-2 and Bad proteins. MCF-7 cells were treated with 100 µg ml^-1^ MLL-2 for different time intervals. (A) Cell lysates were subjected to Western blot analysis for the determination of the expression levels of p53, Bcl-2 and Bad using specific antibodies. (B) The relative densities of each band were expressed graphically by quantitative Western blot analysis in graph (*P<0.05, **P<0.01). High quality figures are available online.

### Effect of MLL-2 on the expression of p53, Bcl-2 and Bad

P53 has been found to be involved in apoptosis induced by a broad range of agents. Western blot analysis was used to see whether MLL-2 has any effect on the expression of this proapoptotic proteins in MCF-7 cells using antibody directed against wild-type p53.

Results in Figure 7 show that 100 µg ml^-1^ MLL-2 treatment, p53 started increasing from as early as 4 hours, reached a maximum level by 12 hours and persisted up to 24 hours. By 48 hours of MLL-2 treatment the level of p53 was found to decrease to control levels. The level of Bcl-2 was moderately low in MCF-7 cells and remained almost unaltered after MLL-2 treatment. On the other hand, the level of Bad increased significantly (P<0.01) after 8 hours and attained a peak at 24 hours after MLL-2 treatment.

### Caspase-3 assay result

**Figure 8.**
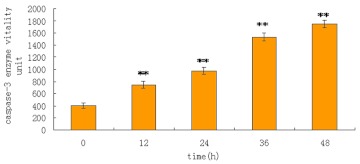
Caspase-3 enzyme vitality in MCF-7 cells treated with MLL-2. Activity of caspase-3 expression in MCF-7 cells after treated with MLL-2. Caspase-3 levels increase with time (**P<0.01). High quality figures are available online.

A specific caspase-3 substrate (Ac-DEVD-*p*NA) was used to estimate the activity of caspase-3 in lysates from MCF-7 cells. Caspase-3-relative activity was significantly increased in the lysates of MCF-7 cells treated with MLL-2 for 48 hours compared with controls (407.4±3.0 vs 1749.2±6.0,P<0.01, Figure 8). Caspase-3 levels increased with treatment time.

## Discussion

Although some studies have tested lectin for potential anti-tumor effects (Franz 1986; Kuttan et al. 1990; Kiss et al. 1997; Pryme et al. 1996; Ryder et al. 1998), no specific result was reported of the effect of lectin purified from *M. domestica* larva. In recent years, studies of the antitumor activities of *M. domestica* have been of particular interest. Studies by Hou's group proved that crude extract from *M. domestica* exhibited antitumor activity, which appeared to be the first evidence that crude extract from *M. domestica* can induce anti-tumor activity in vitro (Hou et al. 2007).

In this paper, M, MLL-1 and MLL-2 from *Musca domestica* larva was purified using affinity chromatography and HPLC. The M, MLL-1, MLL-2 were examined using haemagglutinating and growth inhibition assays against human breast cancer MCF-7 cells, which showed that MLL-2 has significant haemagglutinating activity and an inhibitory effect on MCF-7 cell growth. In this study, MLL-2 was able to inhibit MCF-7 cell proliferation in a dose- and time-dependent manner. However, MLL-2 appears to be less toxic to normal cells. Thus, MLL-2 is a promising candidate as an antitumor agent.

Apoptosis is an active, physiologic form of cell death that is mediated by the internal machinery of certain cells. It is a tightly regulated form of cell death, also called programmed cell death, which is morphologically and biochemically distinct (Reed 2001). Our study demonstrated that MLL-2 induced morphological changes, DNA fragmentation, increase apoptotic rates and arrested cell cycle of MCF-7, which indicated that MLL-2 induced apoptosis in these cells. Morphologically, MCF-7 cells treated with MLL-2 were characterized by chromatin condensation and cell shrinkage shortly after treatment. The nucleus and cytoplasm then fragment, collapse of the nucleus occurs forming small intact fragments (apoptotic bodies) that can be engulfed by phagocytes. Many antitumor agents and DNA-damaging agents arrest the cell cycle and induce apoptotic cell death. The cell cycle checkpoints may ensure that cells have time for DNA repair, whereas apoptosis may eliminate the damaged cells (Klucar et al. 1997; Dirsch et al. 2002; Gamet et al. 2000). Our data demonstrated that MLL-2 had antitumor activity by arresting MCF-7 cells in the G2/M phase and inducing cell apoptosis.

There are three different approaches for inducing apoptosis: the mitochondrial, death receptor, and endoplasmic reticulum pathways. However, the mechanism of MLL-2 action has not been studied in detail. The aim of the experiments was thus to identify the events which may ultimately initiate the apoptotic cascade leading to cancer cell death as a result of MLL-2 treatment.

Distribution of Ca^2+^ is uneven intracellularly. The content of [Ca^2+^]_i_ is higher in mitochondria and endoplasmic reticulum than in cytoplasm and caryon (Woo et al. 2002). Such a concentration gradient between organelle and cytoplasm is a precondition that [Ca^2+^]_i_ can be an intracellular messenger. Slow accumulation of [Ca^2+^]_i_ in mitochondria causes overloading of [Ca^2+^]_i_ so as to open the permeability transition pore, leading to a prompt decrease of the membrane potential (Δ φ m), swelling of mitochondria, and finally apoptosis (Anuradha et al. 2001). In our study, it was found that the [Ca^2+^]_i_ level of the experimental group was significantly higher than that of the control group. Since [Ca^2+^]_i_ is a messenger for the mitochondrial pathway, this indicates that the mitochondrial pathway plays an important role in the process of MCF-7 cells apoptosis.

One of the most interesting questions in the p53 field is how a cell makes the decision to undergo growth arrest or apoptosis. It has been proposed that p53 may induce two sets of genes upon stress signals. One set mainly functions in cell growth control, such as p21/Waf-1 and GADD45, and the other set acts on apoptosis, such as Bcl-2 (Agarwal et al. 1998). In our study, it was observed that MLL-2 is capable of inducing apoptosis in MCF-7 cells, in which expression of p53 can be induced. Apoptosis is accompanied by an increase in levels of p53. It is well recognized that whether a cell becomes committed to apoptosis partly depends upon the balance between proteins that mediate cell death, e.g. Bad, Bax, and proteins that promote cell viability, e.g. Bcl-2 or Bcl-xL (Miyashita et al. 1994; de Aguilar et al. 2000). Interestingly, p53 has been shown to be capable of both down-regulating the death suppressor Bcl-2 and up-regulating the death promoter Bad, thereby changing the Bcl-2/Bad ratio and disposing to programmed cell death. We selected MCF-7 cells in which p53 is constitutively expressed. In these cells, MLL-2 induced apoptosis with an increase in p53 level. Interestingly, the MLL-2-induced increase in p53 expression precedes that of Bad thereby leading us to hypothesize that p53 transactivates Bad expression. In these cells the Bcl-2 level remained almost unchanged thereby shifting the Bcl-2/Bad ratio towards apoptosis. All these observations are evidence for the implication of the p53 signaling pathway in MLL-2 induced tumor cell apoptosis and support the candidature of Bad as the downstream effector in the mitochondrial pathway.

We examined the roles of caspase-3 in MLL-2-induced MCF-7 cells apoptosis. Based on the results of caspase activities, we conclude that caspase-3 played key roles in the post-mitochondrial apoptotic pathway. MLL-2 decreased Bcl-2 expression and activated caspase-3 cascade. Further studies on MLL-2-induced apoptotic signal goes through the caspase pathways in MCF-7 cells are warrented.

Over the years, cancer therapy had witnessed many exciting developments, but the cure of cancer has still remained as complex as the disease itself, since the mechanisms of tumor killing are still not fully realized. Identification of individual components of signaling pathways leading to tumor cell death as well as targeted alteration of those molecules may be of immense help to selectively induce apoptosis in cancer cells. In summary, our study demonstrates that MLL-2 induce MCF-7 cells apoptosis by mitochondrial pathway. Knowledge acquired from this study will, therefore, lead us one step forward towards that goal.

## Abbreviations

AO: acridine orange staining;
HLF: human lung fibroblasts;
MTT: growth inhibition assay;
TUNEL: apoptosis assay
